# Accelerating cine-MR Imaging in Mouse Hearts Using Compressed Sensing

**DOI:** 10.1002/jmri.22718

**Published:** 2011-11

**Authors:** Tobias Wech, Angela Lemke, Debra Medway, Lee-Anne Stork, Craig A Lygate, Stefan Neubauer, Herbert Köstler, Jürgen E Schneider

**Affiliations:** 1 Institute of Radiology, University of WürzburgWürzburg, Germany; 2 Department of Cardiovascular Medicine, University of OxfordOxford, United Kingdom

**Keywords:** mouse heart, infarction, compressed sensing, cine-MRI, cardiac function

## Abstract

**Purpose:**

To combine global cardiac function imaging with compressed sensing (CS) in order to reduce scan time and to validate this technique in normal mouse hearts and in a murine model of chronic myocardial infarction.

**Materials and Methods:**

To determine the maximally achievable acceleration factor, fully acquired cine data, obtained in sham and chronically infarcted (MI) mouse hearts were 2–4-fold undersampled retrospectively, followed by CS reconstruction and blinded image segmentation. Subsequently, dedicated CS sampling schemes were implemented at a preclinical 9.4 T magnetic resonance imaging (MRI) system, and 2- and 3-fold undersampled cine data were acquired in normal mouse hearts with high temporal and spatial resolution.

**Results:**

The retrospective analysis demonstrated that an undersampling factor of three is feasible without impairing accuracy of cardiac functional parameters. Dedicated CS sampling schemes applied prospectively to normal mouse hearts yielded comparable left-ventricular functional parameters, and intra- and interobserver variability between fully and 3-fold undersampled data.

**Conclusion:**

This study introduces and validates an alternative means to speed up experimental cine-MRI without the need for expensive hardware. J. Magn. Reson. Imaging 2011. © 2011 Wiley Periodicals, Inc.

MULTIFRAME MAGNETIC RESONANCE IMAGING (cine-MRI) has become an established tool to assess ventricular volumes and mass in hearts of genetically and surgically modified mice and rats, allowing for a noninvasive characterization of global cardiac function in these models of human cardiac disease. Cine-MRI exams in mice are commonly performed at dedicated small-bore MRI systems equipped with ultrahigh magnetic fields ≥7T, using surface- (1,2), volume-type (3–5) radiofrequency (RF) coils, or a combination of both (6). A stack of short-axis slices is acquired covering the heart from base to apex. Scan times depend on the required temporal and spatial resolution, as well as the available signal-to-noise-ratio (SNR), and typically range from 10–30 minutes. Parallel imaging (PI), and more specifically TGRAPPA (7), has recently been demonstrated to provide a 3–4-fold acceleration of cine-MRI in mice and rats without impacting the accuracy and reproducibility of cardiac functional parameters (8,9). However, PI requires the MR console to be equipped with multiple receiver channels, which are expensive and not always available, particularly on older MR systems. Furthermore, optimized RF coil arrays need to be employed for receiving the MR signal. The dimensions of mice and rats require small array diameters, which represents substantial technical challenges for designing efficient, mutually decoupled coil arrays at ultrahigh magnetic field strengths. Not surprisingly, only very few studies have reported on its use for murine (cardiac) applications so far (eg, 9–13).

Compressed sensing (CS) (14) is a technique that facilitates the reconstruction of signals from a number of acquisitions below the Nyquist limit in any measurement basis if the criteria of sparsity and incoherence are met. While the signal is not required to be sparse in the displayed domain, it is sufficient to know the transformation into a basis, in which the signal has a sparse representation. Due to the time-consuming data acquisition process in MRI and MRS, CS is becoming increasingly more important as a means to reduce acquisition time (15–17). Here, acceleration of the data acquisition process is achieved by sampling a subset of *k*-space followed by a nonlinear reconstruction algorithm (see below).

Few studies have reported on the application of CS in cardiac MRI (18,19), but none in mouse hearts. We therefore sought to investigate the feasibility of compressed sensing as an approach to accelerate cine-MRI in mice at 9.4T and to quantify its impact on global cardiac functional parameters. We demonstrate that up to 3-fold undersampling is generally feasible without impairing accuracy of left-ventricular volumes and mass measurements. Subsequently, accelerated cine-CS-MRI was implemented on the scanner and validated against fully sampled cine datasets.

## MATERIALS AND METHODS

### Animal Preparation

C57Bl/6 mice were obtained from a commercial breeder (Harlan, UK) at least 1 week prior to the MR examination or surgical procedure to allow naturalization to new surroundings. The mice were kept under controlled conditions for temperature, humidity, and light, with chow and water available ad libitum. Five mice (25.9 ± 0.7 g) were subjected to permanent ligation of the left anterior descending coronary artery to generate a chronic myocardial infarction as previously described (20). In brief, the animals were anesthetized with 2% isoflurane in 100% O_2_, intubated, and ventilated with a tidal volume 250 μL and respiratory rate 150/min (Hugo-Sachs MiniVent type 845, Harvard Apparatus, UK). A left thoracotomy was performed in the fourth intercostal space. An intramyocardial suture was placed 1–2 mm below the atrio-ventricular groove with an atraumatic needle and a 6–0 polyethylene suture. Mice were given subcutaneous buprenorphine (0.8 mg/kg) for pain relief. In the validation stage of the project the chronic myocardial infarction group together with matching sham-operated mice (26.6 ± 3.2 g, *n* = 5) were scanned with a standard cine-MRI protocol (5) 8 weeks postsurgery.

To prepare the mice for cine-MRI, anesthesia was induced in an anesthetic chamber using 4% isoflurane in 100% oxygen. Animals were then positioned prone on a warm air blanket (37°C) in the mouse cradle and maintained at 1.5%–2% isoflurane at 2 L/min oxygen flow throughout the MRI experiments. Cardiac and respiratory signals were continuously monitored using an in-house developed ECG and respiratory gating device (21).

All investigations conformed to Home Office *Guidance on the Operation of the Animals (Scientific Procedures) Act*, 1986 (HMSO) and to institutional guidelines.

### Cine-MR Protocol

MRI experiments were carried out on a 9.4T (400 MHz) MR system (Varian, Palo Alto, CA) comprising a horizontal magnet (bore size 210 mm), a VNMRS Direct Drive console, an actively shielded gradient system (1000 mT/m, rise time 130 μs), and a quadrature-driven birdcage resonator (id 33 mm). After positioning the mice in the magnet with the heart in the center, and scouting for long- and short-axis orientation of the heart using a double-gated, segmented gradient-echo (GE) sequence, shimming and pulse calibration were performed automatically prior to each experiment. Eight to nine contiguous, 1-mm-thick slices were then acquired in short-axis orientation covering the entire heart using an ECG-triggered and respiratory gated multiframe sequence with steady-state maintenance during respiration (21). The imaging parameters were: field of view (FOV) 25.6 × 25.6 mm^2^, matrix size 256 × 256, TE/TR = 1.7/4.6 msec, 15° sinc excitation pulse, number of averages NT = 2. The number of frames per cardiac cycle was determined by the heart rate and the number of phase-encoding steps per respiration cycle—acquired in a segmented fashion (5)—was adapted to the respective respiratory rate. Fully sampled data sets only were acquired in sham animals and in mice with chronic myocardial infarction. In addition, accelerated acquisitions (2- and 3-fold) were conducted for a control group (25.8 ± 3.4 g, *n* = 5) using dedicated undersampling patterns for phase-encoding direction as described below.

### CS and the Beating Heart

The theory of compressed sensing and its application to MRI is detailed elsewhere (15,22) and shall only be summarized briefly here. As mentioned above, the successful application of CS has three requirements: 1) transform sparsity, 2) incoherence of undersampling artifacts, and 3) nonlinear reconstruction (22).

Data reconstruction is performed by solving the constrained optimization problem (14):


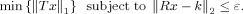
[1]

*T* represents a transformation from complex image space *x* to a sparse representation. *R* denotes a partial Fourier transform corresponding to a given undersampling scheme, *k* are the measured *k*-space data, and ε considers the noise contained in *k*. While minimization of the ℓ_1_-norm promotes sparsity, the additional constraint ‖ċ‖_2_≤ε enforces data consistency (22). In the presented application an altered version of the algorithm by Ma et al. (23) was used for the CS reconstruction, solving the corresponding Lagrangian ℓ_1_-minimization problem:


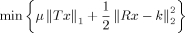
[2]

The parameter μ adjusts the weight of ℓ_1_-minimization relative to the data consistency term, and was set to 0.5 based on empirical studies.

For a dynamic image series {*x*_*i*_}, eg, images of the beating heart, the required sparsity may be found in the temporal domain. Although a single timeframe in the image domain *x*_*i*_ may not be sparse, the content of information in every acquired cardiac phase relative to the temporal average over several timeframes is much lower than the required information for every single frame. Therefore, a difference operator *T* can transform the image series {*x*_*i*_} in a series {*s*_*i*_} with sparse members and is obtained as follows: For each slice, the temporal average image *A* is calculated by summing all cine-frames, and by normalizing to the number of frames *N*, ie:


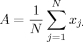
[3]

**Figure 1:**
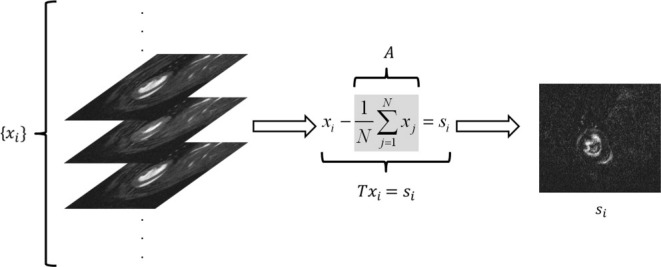
Transformation operator *T*: an average image *A* for the entire image series {*x*_*i*_} is calculated first on a slice-by-slice basis. Sparse representations of individual cardiac phases {*s*_*i*_} are then generated by subtracting the average image from each frame in the cine-train.

Subtracting *A* from each individual image space {*x*_*i*_} leads to a series {*s*_*i*_} of sparse representations. {*s*_*i*_} contains only the temporal information and, thus, meets the CS condition of sparsity (18) (Fig. 1). Having applied the described CS-algorithm on every single element of {*s*_*i*_} *A* is added to each cine-frame again.

### Retrospective Undersampling

**Figure 2:**
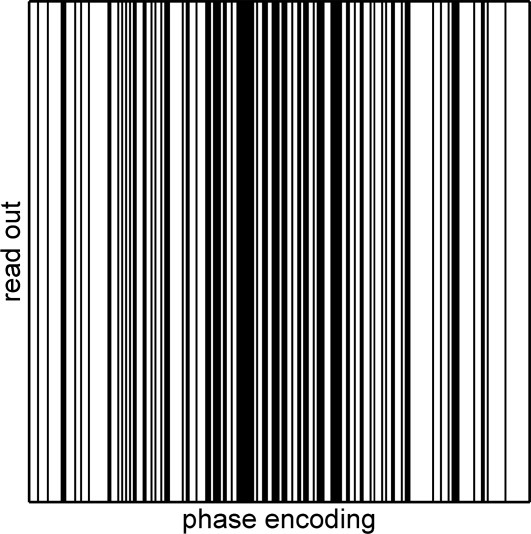
Random sampling scheme for retrospective undersampling. Black lines are acquired, white lines are omitted. The steps are chosen by random numbers according to a Gaussian function with the maximum at the center of *k*-space.

**Figure 3:**
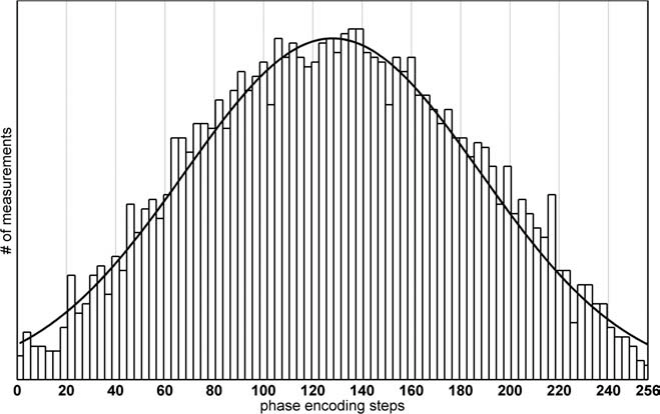
Histogram of all phase-encoding steps over the full cycle of all acquired N timeframes per slice. Each individual sampling scheme represents a random choice of phase encodings steps weighted according to a Gaussian distribution.

In order to investigate the applicability of CS to accelerate cine-MRI in the mouse heart, the *k*-space data for each cine-frame in every slice of the sham and the MI group were retrospectively undersampled in one dimension by a factor of 2, 2.5, 3, and 4, respectively. The sampling scheme was changed for each frame and represented random acquisitions with an additional weight to the center of *k*-space. The patterns for the whole cycle of all acquired cine-frames approximated a Gaussian distribution to realize dense or nearly dense sampling of the low spatial frequency information, which has proven to be beneficial for a successful CS reconstruction (18) (Figs. 2, 3).

### Sampling schemes for In Vivo Study

The point-spread function (PSF) represents a useful tool to determine the coherence of the artifacts caused by a dedicated (random) sampling pattern in *k*-space which is acquired below the Nyquist limit (15). Specifically, the height of the side lobes with regard to the mainlobe of the PSF can be used to determine how localized the artifact energy is (sidelobe to peak ratio (15)). Ideally, the energy is spread evenly across the whole undersampled dimension.

A simulation step was performed prior to the acquisition of real undersampled datasets to determine a suitable series of sampling patterns as a function of the number of timeframes (ie, 20–30, based on the chosen TR and the typical heart rates) in the cine-train per slice, and the degree of undersampling (ie, 2- and 3-fold, following the validation in retrospective undersampling). In all, 200 patterns were created per given number of frames in the cine-train and the according PSFs were determined for the undersampled dimension. The acceleration was limited to the phase-encoding direction and featured random schemes with an additional Gaussian weight to the center of *k*-space as applied in the retrospective studies. The patterns, which were causing the smallest value for the sidelobe to peak ratio (ie, with maximized incoherence for the resulting artifacts) were stored and used for the measurements. Additionally, it was ensured that every phase encoding step was measured at least once over the whole cycle of measurements. Figure 3 shows a histogram of every measured phase encoding step of all timeframes. In this figure the Gaussian character of the *k*-space weighting can easily be seen.

### Data Analysis

Data reconstruction and analysis was performed offline. The undersampled datasets were subjected to the compressed sensing reconstruction outlined above (ie, Eq. [2]), which was implemented in MatLab (MathWorks, Natick, MA), followed by isotropical zerofilling (factor of two), filtering (modified third-order Butterworth filter (24)), Fourier transformation, and the magnitude data were exported into TIFF-format using purpose-written IDL-software (ITT, Boulder, CO). For quantification of cardiac structural (ie, left-ventricular mass, LVM; end-diastolic volume, EDV; end-systolic volume, ESV) and functional parameters, the images (in-plane voxel size 50 × 50 μm^2^) were loaded into Amira 4.1 (Visage Imaging, US) and segmented as described before (5). The operators were blinded to animal ID and acquisition/reconstruction schemes.

### Statistical Analysis

A general linear model analysis for repeated measures was used (SPSS IBM, Chicago, IL) to compare fully and undersampled data obtained in all three groups (ie, MI, sham and control) individually. The Bonferroni correction was applied as a post-hoc comparison of the protocols. A *P*-value < 0.05 was considered statistically significant. Agreement in LV mass, EDV, ESV, SV, and EF between fully and 3-fold undersampled datasets obtained in the sham and the MI mice were graphically assessed using Bland–Altman analysis. Intra- (A.L.) and interobserver (A.L., D.M.) variability were determined in fully and 3-fold accelerated acquisitions in the control group. To test differences between intra- and interobserver variabilities for the two acquisition techniques, Levene's test for equality of variances was used in SPSS.

## RESULTS

**Figure 4:**
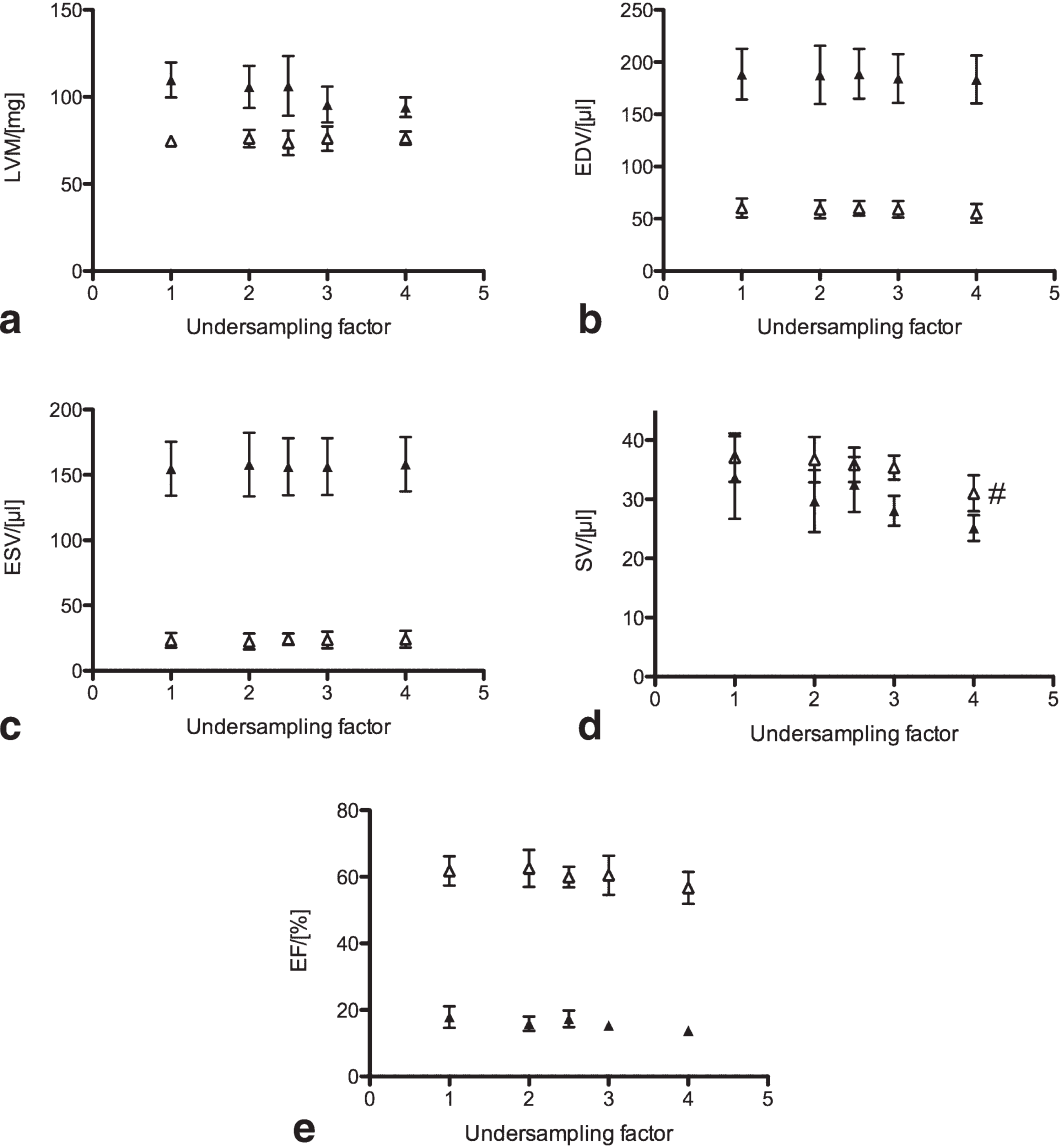
Left-ventricular (**a**) mass, (**b**) EDV, (**c**) ESV, (**d**) SV, and (**e**) EF for sham (open symbols) and chronically infarcted hearts (closed symbols) (mean ± SD, n = 5 each). #*P* = 0.03 between 4- and 2.5-fold undersampled data; *P* = 0.02 between 4- and 2-fold undersampled data; *P* = 0.008 between 4-fold undersampled and fully sampled data.

Cardiac functional parameters, obtained in sham and chronically infarcted mouse hearts from the retrospectively undersampled and CS-reconstructed data, shown in Fig. 4, agreed well between groups. The statistical tests for repeated measures indicate that there is significant variation within the groups for EDV and SV in the sham (*P* = 0.002 and *P* < 0.001), and for EF and SV in chronically infarcted hearts (*P* = 0.013 and *P* = 0.015, respectively). Post-hoc analysis indicated nominal evidence that 4-fold undersampled data were significantly different from the other groups, but this was no longer the case after correcting for multiple testing (Bonferroni), except for SV in sham animals. For this parameter, statistically significant differences were found between 4- and 2.5-fold undersampled data (*P* = 0.03), 4- and 2-fold undersampled data (*P* = 0.02), and 4-fold undersampled and fully sampled data (*P* = 0.008), respectively. The same trend to underestimate SV was observed in infarcted mice, but failed to reach statistical significance, presumably due to higher variability between individuals. Infarct sizes, which were measured in fully sampled datasets only as described in (25), were 44.5 ± 2.5% (min: 40.7%; max: 47.0%).

**Figure 5:**
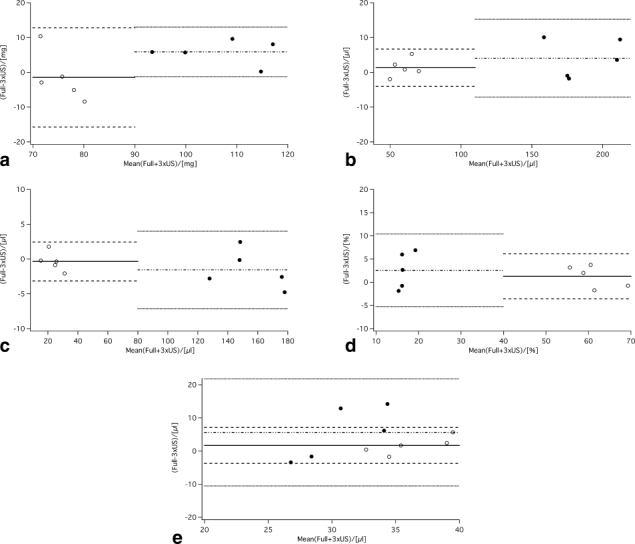
Bland–Altman plots comparing 3-fold undersampled CS data (sham, open symbols; MI, closed symbols) versus standard (ie, fully sampled) acquisition for (**a**) LV mass, (**b**) EDV, (**c**) ESV, (**d**) EF, and (**e**) SV (*n* = 5 each). The solid line indicates the bias for the sham hearts (dashed lines ± 2 SD), and the dashed-dotted line depicts the bias for the MI hearts (dotted lines ± 2SD), respectively.

Figure 5 shows Bland–Altman plots comparing fully versus 3-fold undersampled data acquisition for (a) LV mass, (b) EDV, (c) ESV, (d) EF, and (e) SV for both groups. The bias was 1.5 ± 7.1/5.9 ± 3.6 mg for LV mass, 1.3 ± 2.7/4.1 ± 5.6 μL for EDV, −0.3 ± 1.4/−1.6 ± 2.8 μL for ESV, 1.7 ± 2.7/5.6 ± 8.1 μL for SV, and 1.3 ± 2.4/2.6 ± 3.9% for EF, respectively (sham/MI, mean ± SD, *n* = 5).

**Figure 6:**
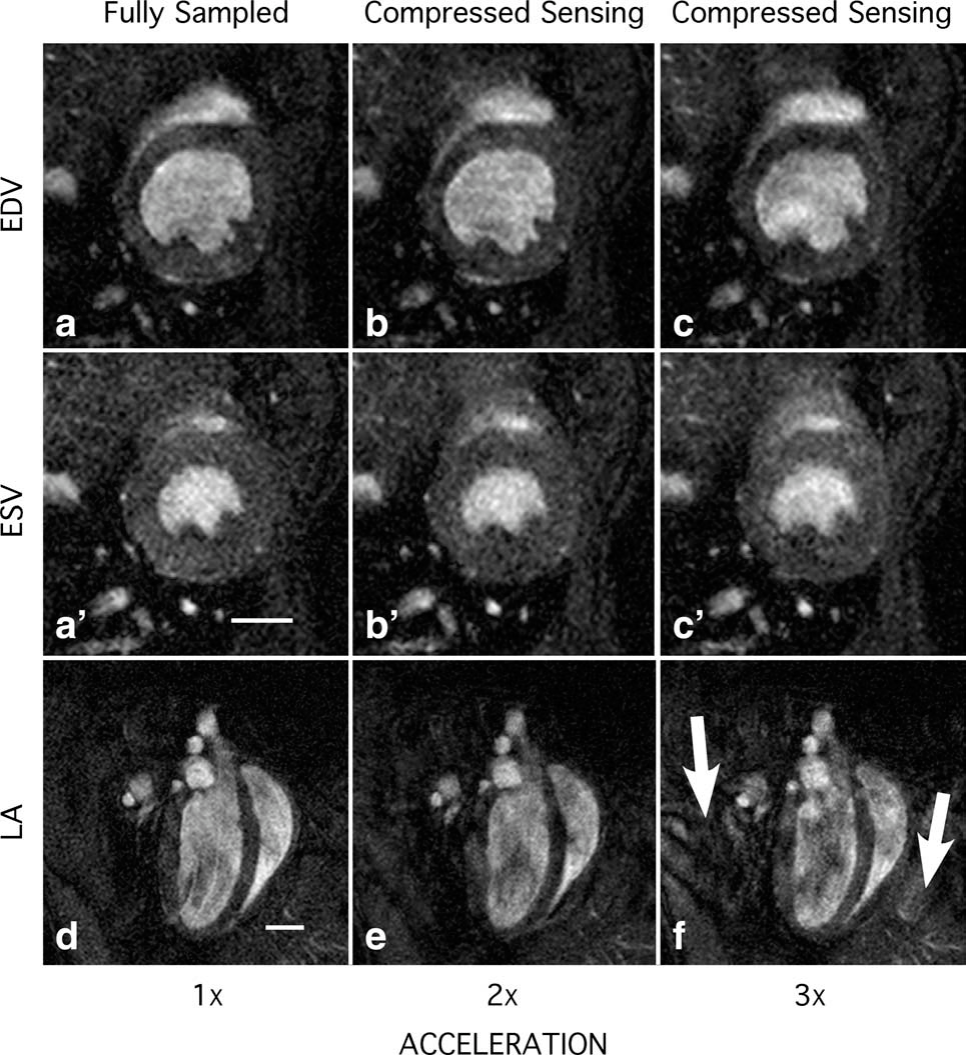
Mid-ventricular short-axis views in (**a–c**) end-diastole and in (**a'–c'**) end-systole; (**d–f**) corresponding end-diastolic 4-chamber-longaxis views. The data were acquired with undersampling factors 1–3. The arrows in panel f indicate an increased artifact in the liver and skeletal muscle. Scale bars = 2 mm.

Figure 6 depicts fully, 2- and 3-fold undersampled mid-ventricular short-axis views in end-diastole (top row, panels a–c) and in end-systole (middle row, panels a'–c'). The bottom row (panels d–f) shows corresponding end-diastolic 4-chamber-longaxis views. The image quality was consistent throughout the entire cardiac cycle (data not shown). The epicardial border appears less well-defined in the 3-fold accelerated datasets (Fig. 6c,c'). An increased artifact level can also be seen in the liver and skeletal muscle of the longaxis view (arrows in 6f). Neither effect, however, impairs quantification of the functional parameters, as demonstrated in Table 1. There were no physiologically relevant differences between fully sampled and 3-fold accelerated acquisition for any parameter. A statistically significant difference was observed for EDV (*P* = 0.018); however, this represents a difference in volume of only 2.1 μL. Furthermore, comparable inter- and intraobserver variabilities were obtained in these datasets (Table 2). Levene's test indicated equal variances between all parameters obtained by accelerated and nonaccelerated data acquisition for intra- and interobserver variabilities, respectively. The scan time decreased from 16.0 ± 0.7 minutes for the fully sampled dataset down to 6.0 ± 0.2 minutes for the 3-fold undersampled data (mean ± SD, *n* = 5).

**Table 1:** Cardiac Functional Parameters Comparing 2- and 3-Fold Undersampled CS Data With Fully Sampled Cine-Acquisition in Control Mice, *n* = 5

	Fully sampled	CS 2x undersampled	CS 3x undersampled
LV mass [mg]	71.5 ± 4.5	71.8 ± 5.9	70.1 ± 2.6
EDV [μl]	54.2 ± 3.7	53.2 ± 3.4	52.1 ± 2.1*
ESV [μl]	22.8 ± 3.7	22.7 ± 3.8	22.8 ± 3.5
SV [μl]	31.4 ± 1.8	30.5 ± 3.3	29.3 ± 2.4
EF [%]	58.2 ± 4.7	57.4 ± 6.1	56.4 ± 5.6

* *P* = 0.018, 3x-undersampled vs. fully sampled data.

**Table 2:** Inter- and Intraobserver Variability

	Fully sampled		CS 3x undersampled	
		
	Intraobserver in %	Interobserver in %	Intraobserver in %	Interobserver in %
LV mass	2.6 ± 2.1	−2 ± 10	2.5 ± 2.1	1.2 ± 5.4
EDV	1.4 ± 1.4	2.9 ± 4.3	1.0 ± 0.8	4.2 ± 5.7
ESV	1.5 ± 1.4	8 ± 12	2.5 ± 1.5	1 ± 15
SV	3.1 ± 1.7	−0.3 ± 7.6	1.3 ± 0.9	7.9 ± 6.3
EF	1.7 ± 1.1	−3.3 ± 7.5	1.3 ± 1.0	3.7 ± 9.2
Overall	2.0 ± 1.6	1.0 ± 9.0	1.7 ± 1.4	3.6 ± 8.7

## DISCUSSION

The aim of this study was to establish and validate CS in preclinical cardiac MRI to accelerate global left-ventricular function imaging in mice without specific hardware requirements. CS has successfully been combined with various MRI and MRS techniques, such as hyperpolarized ^13^C-chemical shift imaging of murine cancer models (26), diffusion tensor imaging of the human brain (27), and human cardiac MRI (18,19,22), to name but a few. However, to the best of our knowledge, CS has not yet been validated for murine cardiac MRI.

To investigate the feasibility of CS in terms of achievable acceleration factors, fully sampled cine data, obtained in sham and chronically infarcted mice, were undersampled retrospectively in postprocessing by randomly omitting phase-encoding steps, prior to implementation on the MR scanner. This approach eliminated the impact of physiological variations during the MR experiments on the analysis of the different datasets. Left-ventricular volumes/mass and ejection fraction, shown in Fig. 4, remain fairly constant up to an undersampling factor of 3. Particularly, statistical testing of the cine data, which were analyzed blinded, indicated nominal evidence that the segmentation results of 4-fold undersampled data were indeed significantly different from the other groups. However, after correcting for multiple testing this was only confirmed for stroke volume in sham animals. On the basis of these results the maximum acceleration factor used in the accelerated acquisitions was therefore set to three. The error bars in Fig. 4 (indicating the standard deviation) are larger for the mouse hearts with chronic infarct due to the inherent heterogeneity of this group. The Bland–Altman analysis (Fig. 5) showed small biases for both groups in all parameters, with a larger spread for the chronically infarcted hearts, where there was a trend to underestimate both LV mass and SV in the 3-fold undersampled datasets. For LV mass, the bias appears systematic but with a low coefficient of repeatability (2× SD), which suggests it may be amenable to application of a correction factor, and should not impact group sizes. In contrast, SV had a relatively high coefficient of repeatability in the 3-fold undersampled datasets (bias: 5.6 ± 8.1 μL), suggesting a slight loss of statistical power for this parameter in the infarcted heart.

Following the initial validation step, implementation at the MR scanner was further optimized by improving the undersampling scheme, which maximized the incoherence of the resulting artifacts and therefore minimized the leakage of energy from the true signal source to other pixels (22). The sampling schemes, which approximated a Gaussian distribution over the whole cardiac cycle, were stored as text-files on the scanner, and chosen during the experiment depending on the observed heart rate (which determined the number of frames in the cine-train) and the selected undersampling factor. This is different from *k-t-*BLAST, where several regularly spaced phase-encode lines are acquired for each frame (28). With additional information acquired during a training phase, *k-t-*BLAST reconstructs images from the undersampled acquisition data (28). Our CS approach yields high quality images. Only minor degradation in image quality can be seen in Fig. 6 for 2-fold undersampling, and, more pronounced, for an undersampling factor of 3. Similar to the retrospectively undersampled data, LV volumes and mass tended to decrease slightly with increasing undersampling factor. This is most likely caused by the reduced edge definition of epi- and endocardial border as can be seen in Fig. 6, rather than by a decrease in SNR. Importantly, CS-reconstruction has been shown to benefit SNR due to its inherently denoising properties (15). Thus, averaging and undersampling are not mutually exclusive as is the case for parallel imaging. Furthermore, experimental reasons can be excluded as all (under-) sampling factors were acquired for each slice, before incrementing the slice offset. However, the observed decrease was only statistically significant for EDV, and was with 2.1 μL between fully sampled and 3-fold undersampled data, which is within the accuracy of the segmentation method. Therefore, it can be considered physiologically irrelevant. Importantly, intra- and interobserver variabilities were not significantly different between these two acquisition protocols.

Our work will benefit experimental cardiac functional MRI, where parallel imaging is not available. In particular, the proposed method provides an alternative means to speed up cine-MRI in mice without the need for dedicated hardware, but with acceleration factors equivalent to those obtained with PI (8,9). Although the data reconstruction was performed offline, the postprocessing time (≈3.5 minutes per slice for 26 cine trains on a 1.66 GHz processor with 2 GB RAM) renders it feasible for online data reconstruction on the MR console computer during the experiment without substantial delay. Increased computational power will additionally reduce reconstruction time. Even though we already discarded the random patterns, which are inappropriate for a CS-reconstruction in a simulation prior to the measurements, the optimization of the *k*-space trajectory can be improved further. Seeger et al (29), for example, proposed a method using a Bayesian inference approximation for an algorithm to optimize sampling patterns dedicated for CS. Furthermore, the current implementation of CS and cine-MRI in this work undersamples in the phase encoding direction only. Undersampling two spatial dimensions (ie, radial sampling patterns, 3D cine-MRI) at once may facilitate higher accelerated acquisitions without significant losses in terms of functional parameter accuracy. However, an appropriate sampling scheme has to be tested and optimized first, too. Finally, expanding CS to *k-t-*space of the cine train by minimizing *x-f-*domain (ie, space-frequency domain) as proposed by Lustig et al (22) as well as transforms that are increasing the sparsity (eg, wavelet transform) could improve the efficiency of the algorithm further.

While the aim of this study was to validate CS as an alternative means to parallel imaging to accelerate cine-MRI in mice, both techniques can also be combined as demonstrated in human hearts (18) to achieve even higher acceleration factors. This is subject of future work, and warrants a systematic investigation of the effect of acceleration provided by each technique on the image quality and subsequently calculated cardiac functional parameters.

In conclusion, CS, in combination with cine-MRI, has been shown to provide a 3-fold acceleration in global cardiac functional imaging in mice at 9.4T, with comparable accuracy in left-ventricular functional parameters, intra- and interobserver variability without specific and (expensive) hardware requirements.
